# Atypical Retinal Phenotype in a Patient With Alström Syndrome and Biallelic Novel Pathogenic Variants in *ALMS1*, Including a *de novo* Variation

**DOI:** 10.3389/fgene.2020.00938

**Published:** 2020-08-21

**Authors:** Laura Mauring, Louise Frances Porter, Valerie Pelletier, Axelle Riehm, Anne-Sophie Leuvrey, Aurélie Gouronc, Fouzia Studer, Corinne Stoetzel, Helene Dollfus, Jean Muller

**Affiliations:** ^1^Service de Génétique Médicale, Institut de Génétique Médicale d’Alsace, Centre de Référence pour les Affections Rares en Génétique Ophtalmologique (CARGO), Strasbourg, France; ^2^Department of Eye and Vision Science, Institute of Life Course and Medical Sciences, University of Liverpool, Liverpool, United Kingdom; ^3^Alder Hey Children’s Hospital Foundation Trust, Members of Liverpool Health Partners, Liverpool, United Kingdom; ^4^Laboratoire de Génétique Médicale, Institut de Génétique Médicale d’Alsace, INSERM U1112, Fédération de Médecine Translationnelle de Strasbourg (FMTS), Université de Strasbourg, Strasbourg, France; ^5^Laboratoires de Diagnostic Génétique, Hôpitaux Universitaires de Strasbourg, Strasbourg, France

**Keywords:** Alström syndrome, *ALMS1* gene, *de novo* variation, high throughput sequencing, retinal dystrophy

## Abstract

Alström syndrome (ALMS) is a rare autosomal recessive multi-organ syndrome considered to date as a ciliopathy and caused by variations in *ALMS1*. Phenotypic variability is well-documented, particularly for the systemic disease manifestations; however, early-onset progressive retinal degeneration affecting both cones and rods (cone-rod type) is universal, leading to blindness by the teenage years. Other features include cardiomyopathy, kidney dysfunction, sensorineural deafness, and childhood obesity associated with hyperinsulinemia and type 2 diabetes mellitus. Here, we present an unusual and delayed retinal dystrophy phenotype associated with ALMS in a 14-year-old female, with affected cone function and surprising complete preservation of rod function on serial electroretinograms (ERGs). High-throughput sequencing of the affected proband revealed compound heterozygosity with two novel nonsense variations in the *ALMS1* gene, including one variant of *de novo* inheritance, an unusual finding in autosomal recessive diseases. To confirm the diagnosis in the context of an unusually mild phenotype and identification of novel variations, we demonstrated the biallelic status of the compound heterozygous variations (c.[286C > T];[1211C > G], p.[(Gln96^*^)];[(Ser404^*^)]). This unique case extends our knowledge of the phenotypic variability and the pathogenic variation spectrum in ALMS patients.

## Introduction

Alström syndrome (ALMS; MIM 203800) is a rare autosomal recessive multi-organ disorder caused by homozygous or compound heterozygous predominantly truncating variations in the *ALMS1* gene, with a prevalence of <1 case per 1,000,000 individuals (Collin et al., 2002; Hearn et al., 2002). The clinical presentation, largely overlapping with ciliopathies, is associated with retinal dystrophy, hearing loss, obesity, insulin resistance, type 2 diabetes, dilated cardiomyopathy, and progressive hepatic and renal dysfunction (Marshall et al., 2005; Mockel et al., 2011). Retinal dystrophy of the cone-rod type is one of the earliest manifestations and presents with photophobia and nystagmus in early infancy (birth to 15 months; Aliferis et al., 2012). It is characteristically rapidly progressive, with blindness by the teenage years. ALMS should be suspected in children with obesity, retinal dystrophy, and dilated cardiomyopathy.

*ALMS1* is composed of 23 exons and encodes a large protein of 4,169 amino acids, initially shown to localize to the centrosome and basal body (Hearn et al., 2005; Knorz et al., 2010) and also perturb actin filament organization (Zulato et al., 2011), suggesting a role in primary cilia function. Although mechanistic details are still lacking, ALMS1 is thought to be implicated in endosomal trafficking, actin organization, maintenance of centrosome cohesion, and transcription (for review, see Hearn, 2019). Clinical diagnosis of ALMS is often challenging in view of the heterogeneity of the clinical phenotype and overlapping features with other syndromes, in particular the Bardet-Biedl syndrome (BBS; MIM 209900) with which it is often misdiagnosed (Aliferis et al., 2012). The retina often manifests disease first, misleading to a diagnosis of congenital achromatopsia, Leber Congenital Amaurosis, or isolated cone-rod dystrophy (Nasser et al., 2018). More than 230 pathogenic variations have been reported so far of which all are truncating variations (Marshall et al., 2015; Astuti et al., 2017). We report here the case of a 14-year-old female who presented with a very mild and unusual retinal phenotype displaying exclusive cone dystrophy with complete preservation of rod function on serial electroretinograms (ERGs), a cardiomyopathy, and a slight, bilateral, and symmetric hearing loss. Two novel class 4 variations (likely, pathogenic) according to the American College of Medical Genetics and Genomics (ACMG; Richards et al., 2015) were identified within *ALMS1*. However, in view of the mild phenotype, the diagnosis was initially questioned. We, therefore, demonstrated that both *ALMS1* variations were *in trans* confirming the biallelic status of the variations. Interestingly, one of the variations occurred *de novo*. The diagnosis of ALMS with the following *ALMS1* genotype was confirmed: c.[286C > T];[1211C > G], p.[(Gln96^*^)];[(Ser404^*^)].

## Clinical Report

A 14-year-old girl born to non-consanguineous healthy French parents presented with central vision loss and photophobia at the age of 6. There were no symptoms of night blindness. Mild high frequency hearing loss was present, associated initially with recurrent otitis media. Past medical history revealed an episode of acute illness aged 1 month caused by hypokinetic cardiomyopathy. Her cardiac disease was stable aged 14 with residual left ventricular dilatation of a non-progressive nature and managed with 5 mg/day of enalapril. Body mass index was normal with no endocrine abnormalities and a normal intellect (Supplementary Table 1).

**Figure 1:**
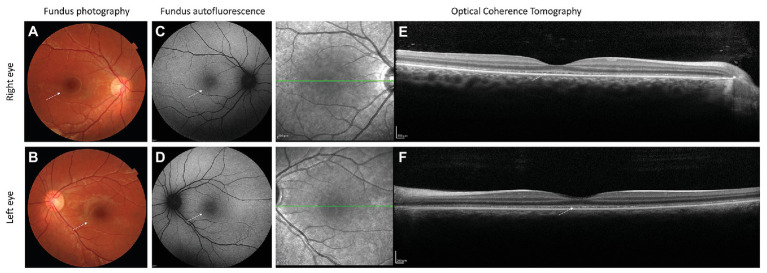
Fundus photography, fundus autofluorescence, and optical coherence scanning images (OCT) of the patient’s retina. Fundus photography of right **(A)** and left **(B)** eyes demonstrates a granular appearance of the macula bilaterally (white arrows). No attenuation of retinal blood vessels can be observed. Fundus autofluorescence of right eye **(C)** and left eye **(D)** demonstrates increased autofluorescence of the fovea bilaterally (white arrows). OCT of right **(E)** and left **(F)** eye shows irregularity of the ellipsoid zone as well as an interdigitation zone in the macular region (white arrows).

**Figure 2:**
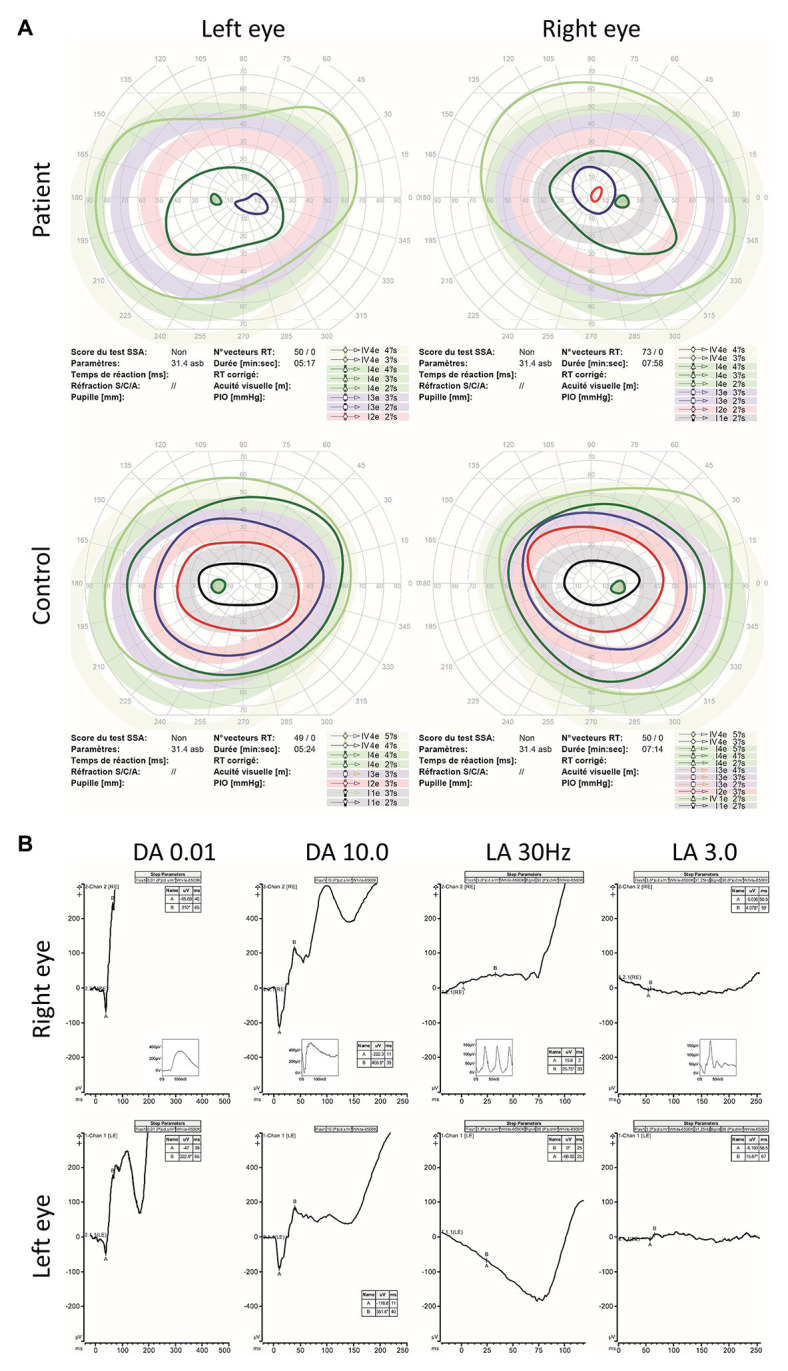
Kinetic visual fields and electroretinogram (ERG) results. **(A)** Kinetic visual fields of the patient’s eyes shows marked central loss of sensitivity with some constriction of the peripheral visual field compared to the reference (control). **(B)** Full field ERG of both eyes were performed according to the ISCEV (International Society for clinical Electrophysiology of vision) standard. Undetectable full field cone ERGs (light adapted LA 3.0 and LA 30 Hz) with subnormal dark adapted (DA 0.01 rod specific) and bright flash dark adapted (DA 10.0) compared to the reference (control is shown as small-integrated panels within each upper panels) is demonstrated.

Aged 14, visual acuity was limited to logMAR 0.60 in both eyes with no nystagmus. Low hyperopia associated with myopic astigmatism was present. Color vision was absent. Fundus examination revealed irregularity of the retinal pigment in the foveal region but normal peripheral retinal appearance, optic discs, and blood vessel caliber (Figures 1A–D). Ocular coherence tomography (OCT) scanning of the macula demonstrated irregularity of the ellipsoid zone, including the photoreceptors bilaterally (Figures 1E,F). Peripheral visual fields demonstrated mild loss of peripheral vision and loss of sensitivity in the central retina (Figure 2A).

ERG was first performed aged 7 and demonstrated cone dysfunction, with complete preservation of rod function. Repeated ERGs aged 9 and 13 confirmed absence of cone responses, with undetectable photopic ERG white flash responses and undetectable 30 Hz flicker responses. However, full dark-adapted rod responses were present, in keeping with an isolated cone dystrophy (Figure 2B). Although, the retinal phenotype was very unusual for ALMS, the diagnosis was considered because of the association of a retinal dystrophy with cardiomyopathy in infancy. Genetic analysis with family segregation was carried out to confirm the diagnosis.

## Materials and Methods

### Patient

The patient was referred for clinical investigations to the Alström National PHRC (“Programme Hospitalier pour la Recherche Clinique”) program held at the reference center for rare genetic eye disorders in the University Hospital of Strasbourg (CARGO, Centre de Référence pour les Affections Rares en Génétique Ophtalmologique). The study protocol had been approved by our Institutional Review Board “Comité Protection des Personnes” (EST IV, N°DC-20142222), and written informed consent was obtained. Our research complied with the Declaration of Helsinki. Best corrected visual acuity (BCVA) was assessed with a retroilluminated decimal Parinaud scale on a Luneau Charts Display L40. Semi-automated kinetic visual fields were performed with the Octopus 900 perimeter (Haag-Streit International, Wedel, Germany). Color vision was assessed with the Farnsworth Panel D-15 color vision cups and Ishihara plates. Slit lamp and dilated fundus examination were performed after pupillary dilatation with tropicamide 1%. Full-field ERG testing was performed with a modified version of the International Society for Clinical Electrophysiology of Vision (ISCEV) protocol under scotopic and photopic lighting conditions using Diagnosys LLC (Lowell, MA, US) device. Both spectral domain OCT and fundus autofluorescence images were acquired using the Spectralis HRA + OCT unit (Heidelberg Engineering, Heidelberg, Germany).

As well as ophthalmological assessment, the patient also underwent audiological and general physical examinations including height and weight with body mass index calculation, pure tone audiograms and review by an otologist, and renal and endocrine assessment by a general physician with measurement of serum urea and creatinine levels, renal echography, fasting glucose and HBA1C levels. Blood samples were obtained using standard venepuncture techniques after an overnight fast.

### Targeted Exome Sequencing

High throughput sequencing of the patient’s sample was performed on the Ion Torrent PGM (Thermofisher) according to the manufacturer’s protocols. DNA libraries were constructed using the HaloPlex Target Enrichment system (Agilent Technologies, version D.5). The libraries were barcoded using HaloPlex ION Barcodes (Agilent Technologies) and then pooled by eight samples. Emulsion PCR was performed on the Ion One Touch 2 system (Life Technologies) and the emulsion PCR products enriched on the One Touch 2 Enrichment System using the Ion PGM Template OT2-200 kit (Life Technologies). Ion sphere particles (ISP) were enriched using the E/S module, charged on one Ion PGM 316 v2 chips and sequenced with an Ion Torrent PGM in a 200-bp configuration run. With an ISP loading of 55%, 149 Mb were produced in total out of 1,264,061 reads, with a median length of 116 bp, of which 12.3 Mb could be used for our patient. Sequencing data were analyzed by the Torrent Suite Software v4.2.1 with alignment to the reference human genome (GRCh37/hg19) and base calling. Variant annotation and ranking were performed by VaRank (Geoffroy et al., 2015) configured with the Alamut batch software (Interactive Biosoftware, Rouen, France). Filtering criteria were applied to identify the disease causing variations, including: (1) removing variants with an allele frequency >1% in public variation databases, such as the 1000 Genomes (The 1000 Genomes Project Consortium et al., 2015) and the ExAC/gnomAD databases (Lek et al., 2016) or our internal patient database (350 samples); (2) removing variants in 5' and 3' UTR downstream and upstream locations, respectively and synonymous variations without pathogenic prediction of local splicing effect. The analysis was focused on compound heterozygous and homozygous variants consistent with a recessive mode of transmission. Each candidate variations were also checked using the Integrative Genomics Viewer (IGV) software (Thorvaldsdóttir et al., 2012). The *ALMS1* nomenclature is based on the RefSeq (O’Leary et al., 2016) accession number NM_015120.4 and the identified variation.

### DNA Analysis (PCR and Sequencing)

**Figure 3:**
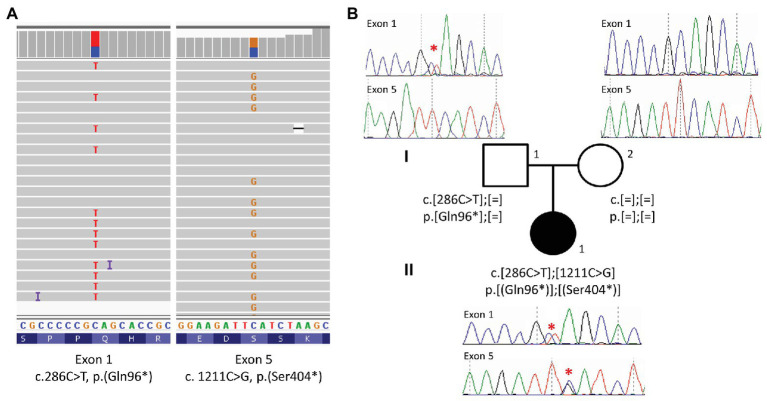
Identification of two pathogenic variations in *ALMS1*. **(A)** Targeted exome sequencing reads aligned to exon 1 and 5 of the *ALMS1* gene, displaying the two heterozygous variations in IGV (Thorvaldsdóttir et al., 2012). **(B)** Pedigree of the family together with Sanger sequencing of exon 1 and exon 5. A red star indicates the variation. The index case (II.1) harbors two nonsense variations in *ALMS1*, the c.286C > T (p.Gln96^*^) is inherited from the father (I.1) and the c.1211C > G (p.Ser404^*^) is *de novo*.

Sanger sequencing was performed using BigDye Terminator V1.1 Cycle Sequencing kit on an ABI3500 (Applied Biosystems, USA), according to the manufacturer’s instructions. Data were analyzed using SeqPilot (JSI Medical Systems, Germany; Figure 3). Primers are available in Supplementary Table 2.

### Cells and RNA Extraction

Total RNA and proteins were obtained from dermal fibroblasts following skin biopsy. RNA was prepared from the cells using a RiboPure kit followed by a DNAse treatment with the TURBO DNA-free. Reverse transcription of 1 μg total RNA to cDNA was performed using the BioRad iScript™ cDNA Synthesis Kit (#170-8891, BioRad, USA).

### RNA Quantification (RT-qPCR)

The qPCR reactions were prepared using the iQ SYBR Green Supermix (#170-8886, BioRad) according to the manufacturer’s instructions using 2 μl of cDNA. Reactions were set up in triplicate (three seperate controls and three separate DNA extractions for the patient). The cycling parameters were as follows: 95°C for 5 min, 45 cycles of 95°C for 15 s, and 60°C for 35 s, followed by the generation of melt curves by heating in 0.5°C increments (5 s/step) for the temperature range 65–95°C. Gene expression levels were quantified relative to the reference genes *GAPDH* and *HPRT* using the efficiency-corrected comparative cycle threshold (C_T_) method using the CFX Manager Software V.1.5 (BioRad). Primers are available in Supplementary Table 2.

### RNA Sequencing

**Figure 4:**
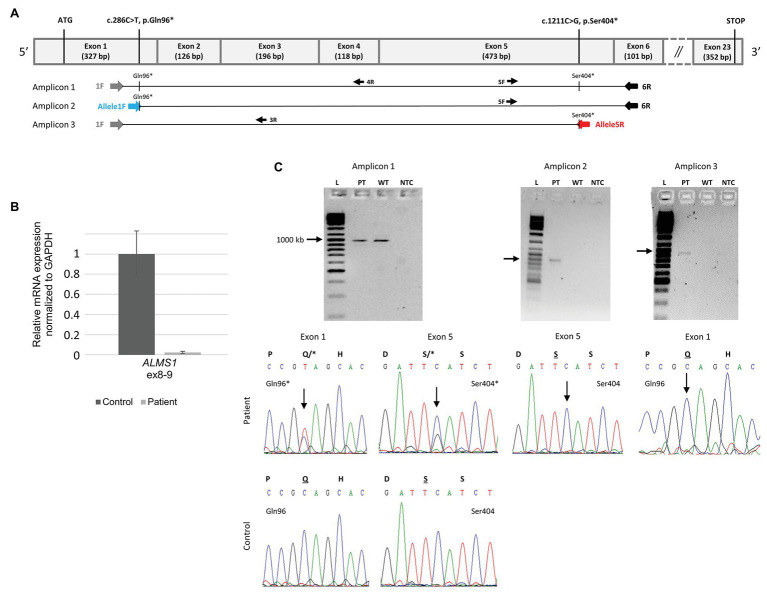
Determining the biallelic status of the two variations using the patient’s RNA. **(A)** Schematic representation of the cDNA of *ALMS1* with exon numbering and size shown. The regions targeted by each amplicon including two allele specific amplicons (Amplicon 2 and 3) together with a single control region (Amplicon 1). The primers targeting each variation are highlighted in blue and red (Supplementary Table 2). **(B)** Real-time quantitative polymerase chain reaction analysis from three technical replicates of II.1 and three controls fibroblasts showed a highly reduced relative expression levels of *ALMS1* (normalized against GAPDH). **(C)** Electrophoresis gels and the corresponding sequences for the three amplicons are shown. Gels are labeled as following: L, Ladder; PT, Patient; WT, wild type control; and NTC, no template control. A single band in each gel and no band in the control for each allele specific (amplicon 2 and 3) demonstrate the specificity of the amplifications. When both variations are on distinct alleles as shown here, each sequence is normal with respect to the other variation.

Three sets of primers (1F-6R for amplicon 1, Allele1F-6R for amplicon 2, and 1F-Allele5R for amplicon 3) were designed from a cDNA sequence according to the RefSeq identifier NM_015120.4 using the Primer3 software^1^ (Figure 4; Supplementary Table 2). PCR reactions were carried out using 50 ng of cDNA, the Taq DNA polymerase (Sigma-Aldrich D4545-5KU), a PCR mix 5X containing a PCR Buffer 10× (100 mM tris-HCl, pH 8.3, 500 mM KCl), a solution of MgCl₂ (25 mM), and the four dNTP (200 μM). PCR conditions were as follow: denaturation at 95°C for 3 min followed by 35 cycles at 94°C for 30 s, 72°C for 30 s, and a final extension of 10 min at 72°C.

The PCR-amplified products underwent electrophoresis on a 1.5% agarose gel. The PCR fragments were purified using the AcroPrep™ 96 Filter Plate 350 μl (Pall Corporation) and sequencing performed by Eurofins Genomics using primers (5F, 4R, and 3R; Figure 4; Supplementary Table 2). Sequenced products were then compared with the *ALMS1* transcript (NM_015120.4) using BioEdit® software and SeqScape Sofware (Applied Biosystems).

## Results

High throughout sequencing of a panel of 31 ciliopathy genes including *ALMS1* (Redin et al., 2012) revealed compound heterozygosity with the identification of two novel rare nonsense variations in the *ALMS1* gene (Figure 3A). The two pathogenic variations include a C > T substitution at nucleotide 286 in exon 1, and a C > G substitution at nucleotide 1,211 in exon 5, resulting, respectively in the replacement of glutamine at position 96 and serine at position 404 by premature stop codons (c.[286C > T(;)1211C > G]; p.[(Gln96^*^)(;)(Ser404^*^)]). Family co-segregation studies confirmed the paternal heritance of the c.286C > T (p.(Gln96^*^)) but no variation could be observed in the mother’s DNA, suggesting that the c.1211C > G (p.(Ser404^*^)) was a *de novo* variation (Figure 3B). However, one cannot exclude a possible *de novo* occurrence on the paternal allele, especially in the context of an unusually mild phenotype. Thus, to confirm the potential consequences of the nonsense variants to the patient’s disease, we showed a highly reduced level of *ALMS1* RNA level (Figure 4B) and demonstrated the biallelic status of the variations using two allele-specific PCR reactions on the patient’s cDNA (Figure 4A), confirming that both *ALMS1* pathogenic variants were indeed *in trans* (Figure 4C). Both variations have never been reported in previous studies (HGMD 2020.1; Stenson et al., 2012), however the c.286C > T is listed in ClinVar under the following identifier RCV000672767.1 (Landrum et al., 2018).

## Discussion

ALMS is a multisystemic autosomal recessive disorder associated with retinal dystrophy of the cone-rod type and severe early onset visual impairment (Marshall et al., 2005; Nasser et al., 2018). Visual symptoms are often the first manifestation of the disease with nystagmus and photophobia in infancy or rarely in the pre-school years (Marshall et al., 2005; Nasser et al., 2018). Our proband presented with early-onset cardiomyopathy, associated with ALMS in 62% of cases, but normally accompanied or closely followed by the development of visual impairment and a cone-rod dystrophy, pointing toward the diagnosis (Marshall et al., 2005). In this case, the late onset of visual symptoms, aged 6, was unusual as early-onset visual difficulties have been reported as universal in ALMS (Russell-Eggitt et al., 1998; Marshall et al., 2005; Xu et al., 2016; Nasser et al., 2018). In the largest reported series of ALMS-related phenotypes (182 cases; Marshall et al., 2005), 98% of patients developed nystagmus and photophobia during the first year of life, with all patients in the series legally blind by the age of 15 (Marshall et al., 2005). However, variability in the severity of the ocular manifestations of ALMS has previously been described (Malm et al., 2008; Nasser et al., 2018). Malm and collaborators reported a patient with a mild ophthalmic phenotype who, aged 10, still had near-normal looking vessels and optic discs, diminished pigmentation of the fundus and visual fields with normal peripheral limits (Malm et al., 2008). However, in this mild case, both cone and rod function was mildly reduced upon ERG testing. A milder ophthalmic phenotype was also reported in a 26-year old female (Nasser et al., 2018) diagnosed with achromatopsia in infancy due to the presence of nystagmus and photophobia at birth, who demonstrated residual vision (20/640 and 20/800, right and left eyes, respectively) in her mid-20s with visual field constriction in the left eye to 20–45 degrees and a remaining crescent of paracentral visual field in the right eye (Nasser et al., 2018). Weiss et al. (2019) also reported a late presentation in a child presenting age 6 with nystagmus and impaired vision diagnosed with Leber Congenital Amaurosis. The child developed systemic features of ALMS (obesity, hypothyroidism, elevated transaminase levels, fatty liver, and acanthosis nigricans) in adolescence, confirming the importance of large-scale genotyping screening (Weiss et al., 2019).

However, while variability in fundus appearance and retinal function assessed by serial ERGs has also been described (Tremblay et al., 1993; Russell-Eggitt et al., 1998; Van den Abeele et al., 2001; Marshall et al., 2005; Malm et al., 2008), the presence of an exclusive cone dystrophy with complete preservation of rod function upon ERG testing in the early teenage years, as in our case, has, to our knowledge, not been previously reported.

The extent and progression of cone-rod dystrophy is an important prognostic feature for the quality of life of ALMS patients, and whilst the full-field ERG may be quite similar to cone-rod dystrophies of other types, the progression of the retinal dystrophy in ALMS is usually very rapid (Russell-Eggitt et al., 1998; Van den Abeele et al., 2001), which has not been the case in our proband. Our case reinforces that the visual outcomes may vary considerably in ALMS.

In addition to the late presentation of first ophthalmic features aged 6, and presence of an exclusive cone dystrophy, our patient also had neither obesity nor insulin resistance/diabetes type 2 (Marshall et al., 2005). Childhood obesity is present in over 95% of individuals with ALMS with hyperinsulinemia in 92% of cases appearing in early childhood. Progression to type 2 diabetes mellitus has been reported in 82% of those older than 16 years (Marshall et al., 2005).

The classical association of ALMS with hypokinetic cardiomyopathy was present, occurring aged 1 month and resulting in stable left ventricular hypertrophy as a consequence. Fortunately, the episode proved non-progressive and without functional consequences, however, she remained on 5 mg of enalapril. Hypokinetic cardiomyopathy is a well know feature of ALMS, with highly variable long-term clinical consequences (Makaryus et al., 2007). In a case series, dilated cardiomyopathy was reported in 62% of patients, with 43% of cases occurring in infancy (age 1 week–16 months; Marshall et al., 2005). In some patients, the infantile cardiomyopathy may be reversible with treatment, but a risk of recurrence remains (Marshall et al., 2005; Makaryus et al., 2007). Our patient also had mild bilateral sensorineural, predominantly high-frequency, hearing impairment evolving throughout childhood. Hearing loss is reported in 89% of patients with a mean age of onset of 5 years (Marshall et al., 2005). Other atypical presentations of ALMS have been reported and include a case of putative isolated cardiomyopathy in a 2-month old child followed up until the age of 4.5 months (Nerakh and Ranganath, 2019). The infant did, however, develop significant weight gain (>95th centile; +3 SD) in the short follow up period (up to 4.5 months) and no formal visual assessments were conducted (Nerakh and Ranganath, 2019).

In this case, molecular analysis confirmed two novel compound heterozygous truncating variations in *ALMS1*. Compound heterozygosity and truncating variations are well described in ALMS, with many affected individuals displaying variations through exons 8 (25%), 10 (27%), and 16 (41%; Aliferis et al., 2012; Marshall et al., 2013). The majority (96%) of the variations are nonsense or frameshift variations (insertions or deletions) leading to premature stop codons producing a truncated protein, or no protein at all (Marshall et al., 2015; Astuti et al., 2017; Chen et al., 2017). We highlight that nonsense-mediated decay (NMD) is likely to occur for our variants as both nonsense variants fall into the NMD-competent regions (NMD+) according to NMDEscPredictor (Coban-Akdemir et al., 2018), a finding corroborated by the very low amount of RNA identified in the patient’s cells.

Given the mild phenotype, one could question whether, although unlikely, the second variation (c.1211C > G, p.Ser404*) that appeared *de novo* could have arisen on the same allele as the first variation. We thus worked on the patient’s fibroblasts and demonstrated the biallelic status of the two variations using allele-specific amplifications. To our knowledge, this is the first description of a *de novo* pathogenic variation in *ALMS1*. *De novo* variants contributing to autosomal recessive diseases are rare, as highlighted by large scale studies of whole exomes on more than 5,000 cases with developmental diseases. These studies did not report a single *de novo* variation contributing to a recessive condition, despite reporting more than 400 autosomal recessive molecular diagnoses (Yang et al., 2014; Retterer et al., 2016). Black et al. (2016) recommended the reporting of *de novo* variations in autosomal recessive disease, as he uncovered two unrelated cases of an autosomal recessive disorder caused by the combination of one *de novo* and one inherited variant in a small series of nine families with severe fetal malformation (Black et al., 2016). According to the literature, the human *de novo* mutation rate ranges from 1.0 to 1.8 × 10^−8^ per nucleotide per generation, leading to <90 *de novo* variants per genome among the 4–5 million variants identified by WGS (for review, see Acuna-Hidalgo et al., 2016). The rate increases with paternal age, and 80% of *de novo* variants are of paternal origin. In our case however, the *de novo* nonsense variant occurred on the maternal allele and the mother’s age at conception was 32. The finding of a *de novo* variant contributing to ALMS in our proband has important implication for genetic counseling, since a clinical diagnosis of ALMS carries a reported recurrence risk of 25%. However, in this case because one of the causative variants arose *de novo*, the recurrence risk is markedly reduced, with significant implications for future reproductive decisions. A small finite risk remains, however, owing to the possibility of germ-line mosaicism in the father/mother. Recurrence risks given by clinical geneticists in such cases are generally stated around 1%. However, one must remain cautious as depending on the gene or disease, parental germline mosaicism levels may be much higher ranging from 5% to as high as 15% (Veltman and Brunner, 2012; Myers et al., 2018).

Our analyses confirmed the biallelic status of the variations in the context of a mild phenotype. To date, contributing factors to phenotypic variability in ALMS remain largely unexplored and may include the presence of modifier alleles and the influence of epigenetic and/or environmental factors. The lifespan of patients with ALMS may not exceed 40 years (Aliferis et al., 2012), however early diagnosis and intervention can moderate the progression of some of the disease phenotypes and improve longevity and quality of life for patients.

## Conclusion

In this unusual presentation of ALMS, we identified two novel pathogenic variations in *ALMS1* that contribute to the high variation load within this gene. This specific case highlights both the genetic and phenotypic variability in ALMS, with an unusually mild retinal dystrophy of the exclusive cone type, a mild cardiac phenotype with absence of endocrine abnormalities, and the presence of a *de novo* variation, a potentially largely overlooked finding in recessive diseases.

## Data Availability Statement

All variants have been submitted to ClinVar (https://www.ncbi.nlm.nih.gov/clinvar/) and can be accessed using the following accessions numbers SCV001142508 and SCV001142509.

## Ethics Statement

Written informed consent was obtained from the minor(s)’ legal guardian/next of kin for the publication of any potentially identifiable images or data included in this article.

## Author Contributions

LM, LP, FS, and VP collected the clinical information. AR, AG, A-SL, and CS collected and analyzed the data (DNA and RNA sequencing). LM, LP, AR, and JM drafted the manuscript. HD and JM supervised and conceived the study. All the authors approved the final version of the manuscript.

### Conflict of Interest

The authors declare that the research was conducted in the absence of any commercial or financial relationships that could be construed as a potential conflict of interest.

## Footnotes

^1^http://frodo.wi.mit.edu/primer3
