# Supplementary material for: Atypical Retinal Phenotype in a Patient With Alström Syndrome and Biallelic Novel Pathogenic Variants in ALMS1, Including a de novo Variation
Source: Front Genet. 2020 Aug 21;11:938. doi: 10.3389/fgene.2020.00938 (PMC7472914; doi:10.3389/fgene.2020.00938)
Supplement: Supplementary file 1 [file Data_Sheet_1.DOCX]

**Supplementary DatA**

| **DEVELOPMENTAL MILESTONES** | **HEARING ASSESMENT**  **(age 11)** | **ENDOCRINE PROFILE**  **(age 13)** | **RENAL PROFILE and LIVER FUNCTION TESTS**  **(age 13)** | **FBC**  **(age 13)** | **CARDIAC ASSESSMENTS** |
| --- | --- | --- | --- | --- | --- |
| Birth weight: 3270 g  Birth height: 50 cm  Gestation: 40 weeks  Independent walking: 14 months  Speech start: 12 months  No sleep disturbance  Normal intellect | Bilateral tympanic membrane dullness  Mild sensorineural hearing loss, predominantly in higher frequencies (2000-4000Hz)  No hearing aids | BMI: 22.6  Weight: 58.5kg  Height: 161 cm  Waist circumference: 72.5 cm  Lipid profile: normal  Pubertal stage: Tanner PIII, start age 11  Normal oestradiol, testosterone, LH/FSH ratios.  Prolactin: 228 mUI/l  Endocrine profile: normal  HBA1C: 5%  ACTH (ng/l): 55.7  Cortisol (ug/l): 128  TSH (mUI/l): 1.71  free T4 (ng/l): 11.2  free T3 (ng/l): 4.65  Leptin (ug/l): 31.8  OGTT: hyperinsulinism without glucose intolerance  Glucose T0 (g/l): 0.88  Insulin T0 (mUi/l): 20.7  Glucose T30 (g/l): 1.8  Insulin T30 (mUi/l): 188  Glucose T60 (g/l): 1.58  Insulin T60 (mUi/l): 180  Glucose T120 (g/l): 1.38  Insulin T120 (mUi/l): 120  HOMA: 0.8 (>3= insulin resistance) | Normal renal function  Urea and electrolytes:  Na^+^ (mmol/l): 138  K^+^ (mmol/l): 4.3  Cl^-^ (mmol/l): 105  Mg^2+^ (mmol/l): 0.83  Urea (mmol/l): 3.6  Creatinine (mmol/l): 44.2  Albumin (g/l): 42  Calcium (mmol/l): 2.34  Phosphate (mmol/l): 1.76  magnesium (mmol/l): 0.83  b2 microglublin (mg/l): 1.73  a1 microglobulin (mg/l): <5.4  Urinalysis:  Albumin/creatinine ratio (mg/mmol): 1.81  Diuresis (ml/24 hours): 700  Creatinine clearance (ml/min): 45  eGFR (ml/min/1.73m^2^): 90  Normal LFTs | Normal | ECG: normal profile  Cardiac doppler US and MRI:  Discreet reduction of left ventricular systolic function.  LEV: 58 % MRI, 50% doppler estimates  Grade I systolic hypertension:  145/85 mmHg |

**Supplementary Table 1**. Synopsis of clinical and biochemical data of the proband. BMI: body mass index. OGTT: oral glucose tolerance test. HOMA: homeostasis model of insulin resistance. LH/FSH: luteinizing hormone/follicle stimulating hormone. HBA1C: haemoglobin A1C. ACTH: adrenocorticotropic hormone. TSH: thyroid stimulating hormone. eGFR: electronic glomerular filtration rate. LFTs: liver function tests. FBC: full blood count. ECG: electrocardiogram. US: ultrasound. MRI: magnetic resonance imaging. LEV: left systolic ejection volume.

| **Application** | **Gene** | **Amplicon name** | **Exon** | **Forward Name** | **Forward (5'-3')** | **Reverse Name** | **Reverse (5'-3')** | **Size (bp)** | **Annealing temperature** |
| --- | --- | --- | --- | --- | --- | --- | --- | --- | --- |
| DNA Sanger | *ALMS1* |  | 1 | Ex1F | AACGTCGCCTGTAGCAAACCT | Ex1R | CTCAGCTGCGCGCGTTTTCT | 647 | 60 |
|  |  |  |  | Ex5F | AGAGTCTGAAATTAGGAGAGCTGT | Ex5R | AGTTCCCTCTTTGTTCCATGGC | 738 | 60 |
| RT-PCR | *ALMS1* | A1 | 1-6 | 1F | TGGACTCCGACTCTCACTACG | 6R | GGCCATCCAGAGTAATGACGTCAGAC | 1125 | 59.8°C |
|  |  | A2 | 1-6 | Allele1F | GATTTTGCCTCCGCTGTCGCCCCaGt | 6R | GGCCATCCAGAGTAATGACGTCAGAC | 1028 | 66.6°C |
|  |  | A3 | 1-5 | 1F | TGGACTCCGACTCTCACTACG | Allele5R | TATGTTTCTGCCTGCTTAGgTc | 1069 | 55°C |
| qRT-PCR | *ALMS1* |  |  | RT-8F | GTGAGGATGGTGGTGGTAGC | RT-9R | TGACCAAATAACTCTGCCTTGA | 247 |  |
|  | *GAPDH* |  |  | RT-4_5F | GGAGCGAGATCCCTCCAAAAT | RT-6R | GGCTGTTGTCATACTTCTCATGG | 197 |  |
|  | *HPRT* |  |  | RT-1_2F | CCTGGCGTCGTGATTAGTGAT | RT-2-3R | AGACGTTCAGTCCTGTCCATAA | 131 |  |
| cDNA Sanger | *ALMS1* |  | 3 | 3R | TCTTCCGTTCTCACATTAAACC |  |  |  |  |
|  |  |  | 4 | 4R | TGAAATAAAGAATCAGGCGCA |  |  |  |  |
|  |  |  | 5 | 5F | TGATGAACTGAAAATTCCCAAA |  |  |  |  |

Supplementary Table 2. List of primers and amplicons used in this study (*ALMS1*: NM_015120.4). The 2 allele specific primers are targeting the specific position of each variation. To ensure the primers correctly amplify only the allele carrying the specific variation it is designed for, one destabilizing change was also incorporated towards the end of the primer. The modified bases are indicated as lowercase red letters.
